# Angiographic and Electrocardiographic Characteristics of Patients With Non–ST-Segment Elevation Myocardial Infarction and COVID-19

**DOI:** 10.1016/j.jscai.2026.105326

**Published:** 2026-04-24

**Authors:** Chase J. Ellingson, Jyotpal Singh, Rosa M. Marticorena, Kevin R. Bainey, G.B. John Mancini, Talha Rafiq, Cesar G. Ginocchio, Jay Parikh, Harene Buvanesan, Joy A. Gatmaitan, Keying Xu, Timothy D. Henry, Payam Dehghani, Shy Amlani

**Affiliations:** a College of Medicine, University of Saskatchewan Regina Campus, Regina, Saskatchewan, Canada; b Prairie Vascular Research Inc, Regina, Saskatchewan, Canada; c William Osler Health System, Brampton, Ontario, Canada; d Canadian VIGOUR Centre, University of Alberta, Edmonton, Alberta, Canada; e University of British Columbia, Vancouver, British Columbia, Canada; f McMaster University, Hamilton, Ontario, Canada; g Unity Health, Toronto, Ontario, Canada; h University of Calgary, Calgary, Alberta, Canada; i Strategic Partnerships in Health Excellence, Research, and Engagement, Li Ka Shing Knowledge Institute, Unity Health Toronto, Toronto, Ontario, Canada; j The Christ Hospital, Cincinnati, Ohio

**Keywords:** acute coronary syndrome, coronavirus, COVID-19, myocardial infarction, non–ST-segment elevation myocardial infarction

## Abstract

**Background:**

COVID-19 may contribute to worse outcomes in acute coronary syndromes through exaggerated inflammation, platelet activation, and endothelial injury, creating a prothrombotic environment. Mortality in ST-segment elevation myocardial infarction (STEMI) with concurrent COVID-19 is markedly higher than in prepandemic cohorts. We characterize patients with non–ST-segment elevation myocardial infarction (NSTEMI) and COVID-19 using core laboratory adjudicated data.

**Methods:**

We conducted a retrospective analysis of patients with NSTEMI and laboratory-confirmed COVID-19 from the William Osler Health System. Coronary angiograms and electrocardiograms were evaluated by the independent core laboratories.

**Results:**

Overall, 155 patients (29.7% women, median age 67 years) were included. In-hospital mortality was 8.4%. In total, 67 patients underwent percutaneous coronary intervention, with 22.3% being unsuccessful. High thrombus grade (3-5) at presentation was observed in 43.3% of patients, and among those with a prior stent, 25% had stent thrombosis. Global systolic dysfunction occurred in 32.3% of patients. Electrocardiograms demonstrated diffuse, nonlocalizing ST changes, with a high prevalence of ST elevation in aVR (33%), conduction abnormalities (19.4%), and Q waves (16.9%), suggesting diffuse thrombotic disease.

**Conclusions:**

This is the first core laboratory description of NSTEMI patients with COVID-19. Compared with prepandemic NSTEMI cohorts, these patients experience higher mortality and lower procedural success rates, likely driven by diffuse thrombotic disease.

## Introduction

The COVID-19 pandemic significantly increased global morbidity and mortality.^1^ Respiratory infections, including COVID-19, can precipitate acute coronary syndrome (ACS) by creating a prothrombotic environment^2^ and causing direct myocardial injury.^3^, ^4^, ^5^ In a large United States ST-segment elevation myocardial infarction (STEMI) cohort, in-hospital mortality was higher in COVID-19–positive patients (n = 551, 15.2%) compared to matched COVID-19–negative patients (n = 2755, 11.2%).^6^

Patients from the North American COVID-19 Myocardial Infarction (NACMI) registry^7^ with STEMI and concurrent COVID-19 experienced markedly elevated in-hospital mortality rates (33%) compared to matched controls (4%).^8^^,^^9^ Patients with COVID-19 and non–ST-segment elevation myocardial infarction (NSTEMI) represent a less studied cohort. In a European COVID-19 ACS registry of 265 patients, including 144 with STEMI and 121 with non–ST-segment elevation ACS (NSTE-ACS), in-hospital mortality for the STEMI subgroup was 22.9% vs 5.7% in controls.^10^ Among NSTE-ACS participants, in-hospital mortality was 6.6% compared to 1.2% in the control group.^10^ Like other viral illnesses, COVID-19 is believed to induce a prothrombotic state characterized by inflammation, endothelial injury, and platelet activation, which promotes both microthrombi and macrothrombi within the coronary circulation.^3^

Despite these findings, research on COVID-19 and NSTEMI remains limited. To the best of our knowledge, no study has used core laboratory adjudicated data to describe the angiographic and electrocardiographic (ECG) characteristics of this population. To address this gap, we used core laboratory data to characterize angiographic characteristics, coronary intervention success rates, clinical outcomes, and ECG findings in patients with NSTEMI and COVID-19.

## Methods

### Patients

Retrospective data collection was completed on adults aged ≥18 years who were identified as having NSTEMI, as per the fourth universal definition of myocardial infarction (MI),^11^ and confirmed SARS-CoV-2 infection (determined by any commercially available test administered during or within 4 weeks before the index hospitalization for MI). All patient data were collected from Meditech at William Osler Health System from April 2020 until December 2023. This study was approved by the William Osler Research Institute’s Research Ethics Board and the Ministry of Health for access to the Ontario Health Data Platform. We compared our COVID-19 NSTEMI cohort to absolute values drawn from previously published, prepandemic NSTEMI data^12^, ^13^, ^14^ and a prior NACMI STEMI angiographic analysis^15^ (Central Illustration).^12^, ^13^, ^14^, ^15^, ^16^

**Central Illustration:**
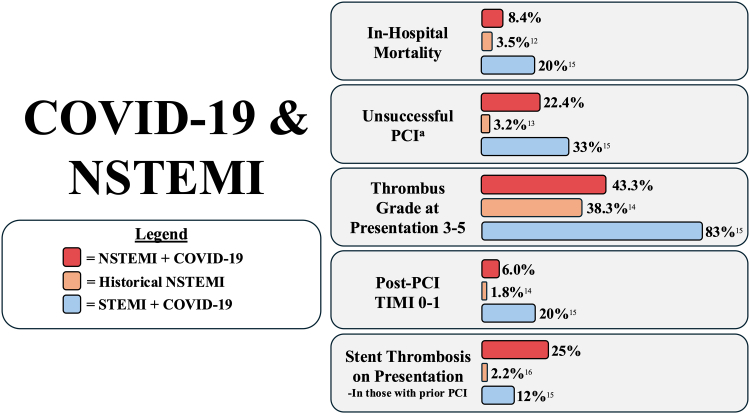
Key differences in COVID-19 non–ST-segment elevation myocardial infarction (NSTEMI) compared with absolute values from historical NSTEMI and COVID-19 ST-segment elevation myocardial infarction (STEMI) cohorts.^12^, ^13^, ^14^, ^15^, ^16^^a^Unsuccessful PCI = TIMI flow <2, diameter stenosis >50%, unsuccessfully treated complication. Note: No formal comparative analysis was conducted between the cohorts in this figure; absolute values from previously published literature were gathered to highlight the unique characteristics of our cohort. No direct comparator for historical NSTEMI cohorts was available for stent thrombosis on presentation; the value here is the rate of stent thrombosis at 1-year post-PCI in STEMI patients. PCI, percutaneous coronary intervention; TIMI, Thrombolysis in Myocardial Infarction.

### Angiograms

Patient index angiograms were sent to the Cardiovascular Imaging Research Core Laboratory (Vancouver, Canada), with angiographic analysis completed as per the NACMI cohort.^15^ Assessment of thrombolysis in myocardial infarction (TIMI) flow, myocardial blush grade (MBG), and thrombus grade burden (TGB) was documented in all patients preintervention and postintervention. Percentage diameter stenosis (DS) via quantitative coronary angiogram of the worst focal lesion in each coronary artery and left main (LM) was performed using edge detection methods (QAngio XA 8.1.2.4, Medis Medical Imaging Systems, Inc). Left ventriculography was completed for assessment of wall motion abnormalities and ejection fraction.

All definitions were consistent with the previously published NACMI angiographic analysis.^15^ Briefly, high thrombus burden was defined as TGB ≥3 in any arterial segment. Stent thrombosis was defined as the presence of any thrombus in the region of the stent, or 5 mm proximal or distal to the stent. For per-patient analysis, arterial segments with the worst (highest) DS, the highest TGB, and the lowest TIMI/MBG were identified. Outcomes of percutaneous coronary intervention (PCI) were classified as: (1) successful if there was TIMI 3 flow, residual stenosis <20%, and no complications/thrombus-related complications; (2) partially successful if there was TIMI 2 flow, residual stenosis 20% to 50%, and/or complications that were successfully treated; and (3) unsuccessful if there was TIMI <2 flow, residual stenosis >50%, and/or a complication that was not successfully treated.

Procedural complications included abrupt closure of the coronary artery, contrast in the pericardium, catheter thrombus, LM dissection, coronary perforation during PCI, distal embolization, new angiographic thrombus, target vessel dissection, LM thrombus, and no reflow phenomenon at any time during PCI.

### Electrocardiograms

The index (diagnostic) ECG were evaluated centrally at the ECG core laboratory at the Canadian VIGOUR Centre (Edmonton, Alberta, Canada), without knowledge of procedural results or clinical outcomes. For complete details on the assessment of ECG, please review Bainey et al^17^ (2026). Briefly, for all patients, the ST elevation (ST-E) was measured at the J point with electronic calipers to the nearest 0.05 mV. ST-E sums (∑ST-E) were calculated as follows: for anterior infarction, the sum of ST-E in V1 to V6, I, and aVL; for inferior infarction, the sum of ST-E in leads II, III, aVF, V5, and V6. ST-segment depression was measured at the J point by similar methods. ST-E in aVR was assessed, given its prognostic value in prior NSTEMI cohorts.^18^ Finally, ST-segment deviation sums (∑ST-D) were calculated by adding the sum of ST-segment depression measured in reciprocal leads to ∑ST-E.^17^ Given the strong predictive value of the sum ST deviation for mortality in STEMI and COVID-19,^17^ we decided to conduct a similar analysis to determine whether it is associated with mortality in our NSTEMI cohort.

## Results

### Participant characteristics

Overall, 155 patients with NSTEMI and COVID-19 (29.7% women, median age 67 years) were included in this study (Table 1). Most patients (71%) were enrolled during 2021 and 2022. Race/ethnicity was as follows: South Asian (70.4%), White (19.4%), Black (6.1%), and East Asian (3.0%). Of the study group, 72.3% had hypertension, 67.7% had dyslipidemia, 52.9% had diabetes, 38.7% had a history of coronary artery disease, 28.4% underwent previous PCI, 23.9% had previous MI, and 13.5% had previous coronary artery bypass grafting. Only 11.6% and 9.0% were current and former smokers, respectively. In our cohort, 82.6% of participants received a COVID-19 vaccine; we do not have details on when the vaccine was administered relative to their hospitalization. The most common treatment strategy was PCI (n = 87, 56.1%), followed by medical management (n = 46, 29.6%) and coronary artery bypass grafting (n = 22, 14.2%). In-hospital mortality occurred in 13 patients (8.4%; 95% CI, 4.5-13.9). No patients experienced in-hospital reinfarction or stroke.

**Table 1:** Participant demographic characteristics

Characteristics	N = 155
Sex	
Female	46 (29.7%)
Male	109 (70.3%)
Age, y	67 (58-75)
Race/ethnicity	
South Asian	69 (70.4%)
White	19 (19.4%)
Black	6 (6.1%)
East Asian	3 (3.0%)
Middle Eastern	1 (1.0%)
History of CAD	60 (38.7%)
Previous PCI	44 (28.4%)
Previous MI	37 (23.9%)
Previous CABG	21 (13.5%)
Hypertension	112 (72.3%)
Dyslipidemia	105 (67.7%)
Diabetes	82 (52.9%)
Previous stroke/TIA	8 (5.2%)
Smoker status	
Current	18 (11.6%)
Former	14 (9.0%)
No	123 (79.4%)
History of CHF	24 (15.5%)
Other medical history	86 (55.5%)
Mechanical ventilation	5 (3.2%)
Mechanical circulatory support	0 (0%)
Symptoms	
Chest pain	94 (60.6%)
Dyspnea	65 (41.9%)
Dyncope	7 (4.5%)
Infarct location	
Anterior	1 (0.6%)
Anterior and inferior	1 (0.6%)
Anterior and posterior	1 (0.6%)
Inferior	1 (0.6%)
Lateral	0 (0%)
NA	151 (97.4%)
Cardiac arrest on presentation	4 (2.6%)
Cardiogenic shock on presentation	1 (0.6%)
Time to patient on table, h	12.40 ± 5.35
Oxygen saturation, %	95.62 ± 4.24
Respiratory rate	18.45 ± 3.52
COVID-19 vaccination	128 (82.6%)
Reperfusion strategy	
CABG	22 (14.2%)
Medical therapy	46 (29.6%)
PCI	87 (56.1%)
In-hospital reinfarction	0 (0%)
In-hospital stroke	0 (0%)
In-hospital death	13 (8.4%)
No. of stents	
0	75 (48%)
1	24 (15%)
2	30 (19%)
3	19 (12%)
4	5 (3%)
5	2 (1%)
Year of admission	
2020	18 (11.6%)
2021	42 (27.1%)
2022	68 (43.9%)
2023	26 (16.8%)
2024	1 (0.6%)
Quarter of admission	
Q1	46 (29.7%)
Q2	41 (26.5%)
Q3	28 (18.1%)
Q4	40 (25.8%)

Values are mean ± SD or median (IQR) for continuous variables and n (%) for categorical variables.

CABG, coronary artery bypass grafting; CAD, coronary artery disease; CHF, congestive heart failure; MI, myocardial infarction; NSTEMI, non–ST-segment elevation myocardial infarction; PCI, percutaneous coronary intervention; Sx, symptoms; TIA, transient ischemic attack.

### Angiographic data

One hundred and twenty-four patients had angiograms interpreted by the study core laboratory (Tables 2 and 3). Forty patients (32%) had identifiable culprit lesions; 5 patients presented with multiple culprit lesions, 35 had a single culprit lesion, and the remaining 84 patients had no angiographically identifiable culprit vessel. Of the identified culprit lesions, the distribution was as follows: right coronary artery/posterior descending artery 46.7%, left anterior descending artery/diagonal branch 31.1%, left circumflex artery/obtuse marginal/posterior descending artery 20.0%, and ramus intermedius 2.2%.

**Table 2:** Key angiographic metrics in COVID-19 and NSTEMI patients

Characteristics	Presentation (n = 124)	Postintervention (n = 67)
DS >70%	72 (58.1%)	6 (9.0%)
DS >20%	92 (74.2%)	40 (59.7%)
High thrombus burden	59 (47.6%)	8 (12.5%)
TIMI 0/1	34 (27.4%)	4 (5.9%)
TIMI <3	47 (37.9%)	11 (16.7%)
MBG 0/1	32 (25.8%)	9 (13.4%)
MBG <3	36 (29.0%)	11 (16.9%)
MV thrombotic disease	12 (9.7%)	0 (0%)
No. of complications	–	5 (7.7%)

Values are n (%).

DS, diameter stenosis; MBG, myocardial blush grade; MV, mitral valve; NSTEMI, non–ST-segment elevation myocardial infarction; TIMI, Thrombolysis in Myocardial Infarction.

**Table 3:** Angiographic parameters in COVID-19 and NSTEMI patients pre-PCI and post-PCI

Parameters	Pre-PCI	Post-PCI
TIMI		
0	11 (16.4%)	3 (4.5%)
1	4 (6.0%)	1 (1.5%)
2	7 (10.4%)	7 (10.6%)
3	45 (67.2%)	55 (83.3%)
N	67	66
Blush		
0	14 (20.9%)	9 (13.8%)
1	0 (0%)	0 (0%)
2	2 (3.0%)	2 (3.1%)
3	51 (76.1%)	54 (83.1%)
N	67	65
Thrombus		
0	23 (34.3%)	49 (75.4%)
1	15 (22.4%)	8 (12.3%)
2	0 (0%)	0 (0%)
3	5 (7.5%)	2 (3.1%)
4	13 (19.4%)	2 (3.1%)
5	11 (16.4%)	4 (6.2%)
N	67	65

Values are n (%).

NSTEMI, non–ST-segment elevation myocardial infarction; PCI, percutaneous coronary intervention; TIMI, Thrombolysis in Myocardial Infarction.

At presentation, 58.1% of participants had >70% DS, 47.6% had high thrombus burden, 27.4% had TIMI 0 to 1 flow, and 25.8% had MBG of 0 to 1. Stent thrombosis was present in 25% of participants with prior PCI at presentation. Altogether, 68 participants underwent PCI; however, one participant’s data could not be interpreted because of a PCI run time issue, leaving 67 participants for the post-PCI analysis. Postintervention, 59.7% had >20% DS, 12.5% had high thrombus burden, 16.7% had TIMI <3, 16.9% had MBG <3, and 7.7% had complications. Coronary intervention was deemed successful, partially successful, and unsuccessful in 41.7%, 35.8%, and 22.4% of participants, respectively (Table 4). The most common reasons for unsuccessful coronary intervention were post-PCI thrombus grade 3 to 5/thrombus-related complications (88.2%), >50% DS (85.7%), and TIMI 0 to 1 flow (28.6%).

**Table 4:** Coronary intervention success rates in NSTEMI and COVID-19

	Overall (n = 67)	Unsuccessful (n = 15)	Partially successful (n = 24)	Successful (n = 28)
TIMI				
0/1	4 (6.1%)	4 (28.6%)	0 (0%)	0 (0%)
2	7 (10.6%)	3 (21.4%)	4 (16.7%)	0 (0%)
3	55 (83.3%)	7 (50.0%)	20 (83.3%)	28 (100%)
DS				
<20	29 (40.3%)	1 (6.7%)	1 (4.2%)	27 (96.4%)
20-50	24 (37.3%)	1 (6.7%)	22 (91.7%)	1 (3.6%)
>50	13 (22.4%)	12 (85.7%)	1 (4.2%)	0 (0%)
Target vessel dissection	1 (1.5%)	1 (6.7%)	0 (0%)	0 (0%)
Thrombus-related complications	4 (6.0%)	4 (26.7%)	0 (0%)	0 (0%)
High thrombus (3-5)	8 (12.5%)	8 (61.5%)	0 (0%)	0 (0%)
In-hospital mortality	3 (4.5%)	0 (0%)	0 (0%)	3 (10.7%)

Values are n (%). All values, except TIMI and DS, correspond to post-PCI. One patient can have more than 1 outcome; therefore, 1 patient may appear in more than 1 category for each variable. For TIMI, low score dominants; for DS, high score dominants. High thrombus was defined based on the maximum thrombus value of each patient. Thrombus-related complications include distal embolization and new angiographic thrombus.

DS, diameter stenosis; NSTEMI, non–ST-segment elevation myocardial infarction; TIMI, Thrombolysis in Myocardial Infarction.

Among the 92 participants who underwent left ventriculography (Table 5), the median ejection fraction was 47.70%. Global LV dysfunction was found in 32.3% of participants. Segmental LV dysfunction was present in 56.9%, with anterior, inferior, and apical wall motion abnormalities observed in 64.6%, 61.6%, and 61.5% of participants, respectively.

**Table 5:** Left ventriculography in patients with COVID-19 and NSTEMI

	NSTEMI (n = 92)
Ejection fraction	47.70 (39.95-53.68)
Left ventricular description	
Global dysfunction	21 (32.3)
Normal	6 (9.2)
Segmental wall motion abnormality	37 (56.9)
Takotsubo	1 (1.5)
Anterior wall motion	
Akinetic	6 (9.2)
Dyskinetic	0 (0.0)
Hypokinetic	36 (55.4)
Normal	23 (35.4)
Inferior wall motion	
Akinetic	7 (10.8)
Hypokinetic	33 (50.8)
Normal	25 (38.5)
Apical wall motion	
Akinetic	9 (13.8)
Dyskinetic	10 (15.4)
Hypokinetic	21 (32.3)
Normal	25 (38.5)

Values are median (IQR) for continuous variables and n (%) for categorical variables. LV, left ventricle; NSTEMI, non–ST-segment elevation myocardial infarction.

### ECG data

Complete ECG data are available in Table 6. Of all participants, only 6 had ECG localizing to a particular coronary territory (3.9%), suggesting diffuse ST-segment changes that were not concordant with the infarct distribution. Normal sinus rhythm was noted in 94.8% of participants, and 3.9% had atrial fibrillation. Furthermore, conduction abnormalities and Q waves were frequently present in 19.4% and 16.9% of patients, respectively. Median sum ST deviation was 3.4 mm (1.6-5.3), and univariable analysis did not demonstrate an association between the sum ST deviation and mortality (relative risk [RR] 1.06; 95% CI, 0.92-1.17). ST-E in lead aVR was noted in 48 participants (33%), with a median ST-E of 0.5 mm (0.3-0.7); when investigating ST-E in aVR and mortality, an RR of 1.88 (95% CI, 0.53-5.88) was found.

**Table 6:** Electrocardiographic features of patients with NSTEMI and COVID-19

	N = 155
Sinus rhythm	147 (94.8%)
SVT	1 (0.6%)
Atrial fibrillation	6 (3.9%)
First-degree AVB	8 (5.2%)
Second-degree AVB	0 (0%)
Third-degree AVB	0 (0%)
Complete LBBB	11 (7.1%)
Complete RBBB	11 (7.1%)
Left anterior hemi-block	1 (0.6%)
Left posterior hemi-block	0 (0%)
LVH (Sokolow-Lyon)	1 (0.6%)
LVH (Cornell voltage)	4 (2.6%)
RVH (Butler-Leggett)	0 (0%)
Low-voltage	0 (0%)
MI location unknown	139 (95.9%)
Index anterior MI (V1-V4)	3 (1.9%)
Index inferior MI (II, III, aVF)	3 (1.9%)
Index lateral MI (I, aVL)	0 (0%)
Index apical MI (V4-V6)	0 (0%)
Index posterior MI (V1-V2)	0 (0%)
Sum ST deviation, mm	3.4 (1.6-5.3)
Q waves	26 (16.9%)
Concave	2 (1.3%)
Horizontal	149 (96.1%)
Shape cannot be analyzed	4 (2.6%)

Values are median (IQR) for continuous variables and n (%) for categorical variables.

AVB, atrioventricular block; LBBB, left bundle branch block; LVH, left ventricular hypertrophy; MI, myocardial infarction; NSTEMI, non–ST-segment elevation myocardial infarction; RBBB, right bundle branch block; RVH, right ventricular hypertrophy; SVT, supraventricular tachycardia.

## Discussion

This is the first study to describe core lab adjudicated angiographic and ECG characteristics of patients with NSTEMI and concurrent COVID-19 (Central Illustration). Several notable findings were observed. First, NSTEMI patients with COVID-19 had an in-hospital mortality rate of 8.4%, more than double that of prepandemic NSTEMI cohorts (3.5% in the SWEDEHEART registry^12^). Second, only 32% had a core laboratory identified culprit vessel (compared to 86% in historical NSTE-ACS data^19^); PCI was performed in 56% of those who underwent angiography. Thrombus was prominent at presentation, with high thrombus grade (3-5) noted in 47.6% of participants; furthermore, 25% of those with prior PCI presented with stent thrombosis. Unsuccessful PCI occurred in 22.3% of cases (driven primarily by thrombus and thrombus-related complications), which is substantially higher than prepandemic NSTEMI (3.2%).^13^ Fourth, only 6 of 155 ECG showed a localizing pattern; the majority had diffuse ST changes, with 33% showing ST-E in aVR and 19.4% showing conduction disturbances, consistent with previously reported multivessel microthrombi.^20^

### Mortality

Among the 155 patients with NSTEMI and COVID-19, in-hospital mortality occurred in 8.4% of cases. Another study demonstrated a 6.6% mortality rate in NSTEMI and COVID-19 patients.^10^ In comparison, among a group of 99718 NSTEMI patients from the SWEDEHEART registry, in-hospital mortality was 3.5%.^12^ Increased mortality among patients with NSTEMI and COVID-19 aligns closely with the work previously reported by Saad et al^6^ (2021), Kite et al^10^ (2021), and the NACMI registry, which found markedly elevated mortality among those with STEMI and COVID-19.^8^^,^^9^ Previous NACMI analyses reported that 12.3% of patients with STEMI and COVID-19 required mechanical circulatory support (MCS); despite similar risk factors, these patients had a higher mortality rate than those not requiring MCS, likely due to extensive pulmonary involvement.^21^ No patients in our cohort received MCS. Increased mortality among COVID-19 patients with NSTEMI may be a manifestation of the inflammatory and prothrombotic state, leading to larger territory infarcts, more inflammation, and accelerated platelet activation.

### Angiographic data

In our NSTEMI cohort, the majority had no identifiable culprit artery (67.7%) compared to only 14% in a prepandemic NSTE-ACS cohort.^19^ High thrombus grade (3-5) was present in 47.6% of our cohort pre-PCI and 12.4% post-PCI. Previous literature reported TIMI thrombus grade 3 to 5 at presentation in 38.3% of non–COVID-19 NSTEMI cohorts.^14^ Surprisingly, among those with prior PCI, stent thrombosis on presentation was observed in 25% of patients. Although comparative stent thrombosis data are unavailable for NSTEMI patients, this rate of 25% is 11-fold higher than the stent thrombosis rate at 1-year follow-up in STEMI patients.^16^ Post-PCI, TIMI 0 to 1 flow was observed in 7.4% of patients. Previous NSTEMI studies have reported post-PCI TIMI 0 to 1 flow in 1.8% of patients.^14^ In addition, high rates of post-PCI TIMI <3 flow and MBG <3 (16.7% and 16.9%, respectively) further suggest diffuse thrombotic disease in our cohort. Unsuccessful PCI occurred in 22.3% of COVID-19 NSTEMI patients, compared to 3.2% reported in the prepandemic literature.^13^ Of the 124 patients with angiograms, 32% of whom had identifiable culprit lesions, relatively few (54%) underwent PCI. Similarly, the fact that 29.6% of NSTEMI and COVID-19 patients received medical management alone further supports the notion of a unique phenotype of predominantly diffuse disease with microvascular thrombi, altered coronary flow, and high thrombus burden. It is important to note that management decisions were determined by the treating physician. Prior studies have demonstrated systematic differences between operator and core laboratory angiographic assessments, with operators tending to rate lesions as more severe before PCI and more favorable after PCI, potentially biasing toward intervention.^22^^,^^23^ This difference may partly explain some of the variation between angiographic findings and management decisions. Similar to STEMI and COVID-19,^15^ we hypothesize that the inflammatory and prothrombotic milieu of COVID-19 contributes to the increased mortality and higher rates of unsuccessful revascularization observed in this population.

On left ventriculography, 32.3% and 56.9% participants had global and segmental systolic dysfunction, respectively, consistent with the diffuse thrombotic disease as demonstrated on angiography and ECG.

### ECG data

Most participants had ECG with diffuse ST changes, not corresponding to the infarct site distribution, with only 6 having localizing ECG. In keeping with diffuse disease, 33% of our population had ST-E in aVR, a predictive factor of LM or 3-vessel coronary artery disease and poor outcomes.^18^ However, in this case, it may have been due to diffuse microvascular thrombi in COVID-19, leading to these global ECG changes. Furthermore, conduction abnormalities were prevalent (19.4%), consistent with a myocardial rather than epicardial issue. A high proportion of Q waves (16.9% at presentation) may reflect the 23.9% of participants with prior MI, though it could also represent more advanced infarcts at presentation. Among NSTEMI and COVID-19 patients, there was no significant relationship identified between the sum ST deviation and mortality (RR, 1.06; 95% CI, 0.92-1.17), but this could be because of the small sample size. When investigating ST-E in aVR and mortality, an RR of 1.88 (95% CI, 0.53-5.88) was found.

### Limitations

Multiple limitations to this work should be considered. First, the small sample size and low absolute number of deaths precluded multivariable analyses of mortality predictors and resulted in wide confidence intervals in univariable analyses. Angiographic analysis has inherent limitations, and research suggests that intravascular imaging can identify culprit lesions in most NSTE-ACS patients without an angiographically identifiable culprit lesion.^19^ Myocardial injury during COVID-19 infection has been shown to occur via multiple mechanisms (MINOCA, myocarditis, and stress cardiomyopathy); transthoracic echocardiography prior to angiography, with a focus on regional wall motion abnormalities, could help differentiate ACS from these other mechanisms.^24^ Other clinical information, including COVID-19 symptoms and specific medications used (for ACS management and COVID-19), was not available. Additionally, the data in this study were collected at a single center, and 70.4% of participants were of South Asian race/ethnicity, limiting the generalizability of the findings. Only 80.0% and 59.4% of the cohort had angiograms and left ventriculography available for core laboratory analysis, respectively. It is important to recognize that the data used for comparisons were from previously published studies, not from COVID-19–negative patients at the same center and time period; this warrants cautious interpretation. Finally, the lack of independent event adjudication may have introduced bias in outcome classification.

## Conclusion

This study is the first to leverage core laboratory interpreted angiograms and ECG data to characterize NSTEMI patients with COVID-19. We found that these patients exhibited diffuse, nonlocalizing ST changes on ECG, along with high thrombus burden, stent thrombosis, and LV dysfunction, while a few culprit lesions were identified on angiogram. They experienced increased mortality and a higher probability of unsuccessful revascularization. These findings mirror prior COVID-19 STEMI research and highlight the prothrombotic and inflammatory milieu associated with COVID-19.

## Declaration of competing interest

The authors declared no potential conflicts of interest with respect to the research, authorship, and/or publication of this article.
